# The Influence of Motive and Outcome on Age-Related Differences in Prosocial Evaluation

**DOI:** 10.1093/geroni/igaf122.1877

**Published:** 2025-12-31

**Authors:** Hongmei Lin, Helene Fung

**Affiliations:** The Chinese University of Hong Kong, Hong Kong, China (People’s Republic); The Chinese University of Hong Kong, Hong Kong, China (People’s Republic)

## Abstract

Prosocial behavior increases with age, but how older (vs. younger) adults’ prosocial behaviors are evaluated remains unclear. Across two pre-registered studies, younger and older participants evaluated prosocial behaviors stemming from various motives (material, social, emotional, other’s benefit) and resulting in different outcomes (positive vs. negative). Study 1, which did not disclose actor’s age, included 155 older (54% female, Mage = 59.90, SDage = 3.88) and 175 younger adults (51% female, Mage = 28.20, SDage = 3.94). Study 2, which identified the actor as either younger or older, included 279 older (55% female, Mage = 59.50, SDage = 4.18) and 340 younger adults (58% female, Mage = 30.40, SDage = 3.43). Both studies revealed that, compared to actions driven by material and social motives with positive outcomes, younger and older participants rated prosocial acts as more prosocial when they are motivated by emotional or other-oriented benefits, even if the outcome was negative. Furthermore, when the actor’s age was disclosed, both older and younger participants applied similar standards in their prosocial evaluations when outcome was positive. However, with negative outcome, older participants showed a bias: they were more lenient towards older actors performing prosocial acts for social benefits compared to younger actors, but less lenient when those acts were performed for material benefits. These findings suggest that emotional value is perceived as a genuine motive for prosocial behavior across ages. Moreover, older adults demonstrate greater flexibility in prosocial evaluations, adjusting their standards based on actor’s age and motives when negative outcomes occur.

